# Physical activity is associated with exercise capacity among patients undergoing chemotherapy: A pilot randomized controlled trial

**DOI:** 10.1371/journal.pone.0329774

**Published:** 2025-09-29

**Authors:** Alexis Antonucci, Alexander R. Lucas, Juliana Costa, Sam Norton, Peter Brubaker, Shannon Mihalko, Alexandra Marshall, Jeremy Via, R. Lee Franco, Amy Ladd, Rakhee Vaidya, Victor Yazbeck, Lynne Wagner, W. Gregory Hundley

**Affiliations:** 1 School of Medicine, Virginia Commonwealth University, Richmond, Virginia, United States of America; 2 Department of Internal Medicine, Division of Cardiology, Pauley Heart Center, Virginia Commonwealth University, Richmond, Virginia, United States of America; 3 Department of Social and Behavioral Sciences, School of Public Health, Virginia Commonwealth University, Richmond, Virginia, United States of America; 4 Department of Health and Exercise Science, Wake Forest University, Winston-Salem, North Carolina, United States of America; 5 Department of Kinesiology and Health Sciences, Virginia Commonwealth University, Richmond, Virginia, United States of America; 6 Department of Hematology and Oncology, Wake Forest University, Winston-Salem, North Carolina, United States of America; 7 Department of Internal Medicine, Hematology/Oncology, Virginia Commonwealth University, Richmond, Virginia, United States of America; 8 Department Health Policy and Management, University of North Carolina at Chapel Hill, Chapel Hill, North Carolina, United States of America; Asian Institute of Medicine Science and Technology: AIMST University, MALAYSIA

## Abstract

**Introduction:**

Exercise intolerance is common among cancer patients undergoing active treatments. We conducted a pilot study in patients receiving potentially cardiotoxic therapies to investigate the impact of participation in a 6-month physical activity intervention (PAI) versus a healthy lifestyle education control intervention (HLI) on accelerometer-derived measures of PA (steps/day or minutes of moderate to vigorous PA [MVPA]/week) and whether these measures were associated with submaximal exercise capacity (6-minute walk distance [6MWD]).

**Methods:**

Participants with breast cancer or lymphoma (n = 33) were randomized (2:1) into PAI or HLI groups. Exercise training was patient-centered, tailored by treatment and functional status. Objective assessments of PA (steps/day, MVPA/week), and submaximal exercise capacity (6MWD) were completed at baseline, 3-, and 6-months. Descriptive statistics were used to examine changes in PA and 6MWD. T-tests were used to examine differences in 6MWD between groups at 3- and 6-months.

**Results:**

At baseline, 27 participants in the PAI (n = 20) and HLI (n = 7) groups who completed baseline and at least 1 follow up visit, had a mean age of 53 (range: 23–76) vs 56 (range: 40–77) years, took on average 8,330 ± 2520 vs 7800 ± 3830 steps/day, did an average of 133 ± 44 vs 109 ± 87 minutes of MVPA/week, and had a mean 6MWD of 496 ± 94 vs 440 ± 18 meters, respectively. At 3- and 6-months there was a 87m and 76m difference in 6MWD by intervention group. A correlation analysis did find significant associations between steps/day (r = 0.51, p = .04) and 6MWD at 3-months.

**Conclusions:**

Baseline data indicate that, on average, our study sample was close to meeting the PA guidelines. Unexpectedly, both groups maintained their activity levels up to 3- and 6-months. Submaximal exercise capacity (6MWD) was also maintained (with a clinically meaningful but not statistically significant relative benefit in the PAI) suggesting higher PA levels may associate with reduced exercise intolerance after anthracycline therapy.

## Introduction

Cardiotoxic cancer therapies, such as anthracyclines, are commonly used for the treatment of breast cancer and lymphoma. Exercise intolerance is a common complication of treatment among patients undergoing anthracycline-based chemotherapy. Individuals diagnosed with breast cancer, throughout the disease continuum, have a decreased cardiopulmonary function as measured by cardiopulmonary exercise testing (VO_2_ peak) which is the gold standard assessment of cardiorespiratory fitness [1].

Additionally, anthracycline-based chemotherapy can have cardiotoxic effects causing left-ventricular dysfunction, which can ultimately progress to congestive heart failure (CHF) [2,3]. Given the potentially cardiotoxic effects of anthracyclines, several recent studies have examined whether exercise interventions during chemotherapy can prevent or limit these cardiotoxic effects. Specifically, a systematic review of 10 studies found that among breast cancer patients undergoing anthracycline-based chemotherapy, left ventricular ejection fraction (LVEF) and cardiorespiratory fitness could be improved or preserved by concurrent moderate-to-high intensity aerobic exercise done continuously or in intervals for more than eight weeks [4].

Given the potential benefits of exercise training during receipt of cardiotoxic therapies, we conducted a pilot study in adults with lymphoma or breast cancer who were receiving potentially cardiotoxic treatments to investigate the impact of a 6-month physical activity intervention (PAI) versus a healthy lifestyle education control intervention (HLI) on accelerometer-derived measures of physical activity (steps/day or minutes of moderate to vigorous physical activity [MVPA]/week) and whether these measures were associated with submaximal exercise capacity (6-minute walk distance [6MWD]). Steps per day were specifically analyzed due to their impact on cardiovascular health; a recent meta-analysis of 8 prospective studies showed that in adults sixty or older, walking more steps per day was associated with having lower cardiovascular disease risk [5]. The study also found that walking between 6,000 to 9,000 steps per day, compared to 2,000 steps per day, was associated with a 40% to 50% reduced risk of cardiovascular disease among adults sixty or older [5].While this study did not directly look at individuals receiving anthracycline-based chemotherapy, the positive impact of walking on cardiovascular health and the feasibility of walking among participants with low functional capacity make it a potentially important component of survivorship care and thus we sought to determine whether steps per day was associated with exercise capacity. We hypothesized that participants in the PAI group would take more steps per day and have an increased MVPA/week in comparison to the HLI group leading to a higher 6MWD.

## Materials and methods

### Study design/participant characteristics and recruitment

From January 2019 to March 2022, we conducted a 6 month, 2-armed, randomized controlled (2:1) pilot-feasibility study: the Physical Activity and Lymphoma study (PALS) (NCT01719562). The study was conducted at Atrium Health Wake Forest Baptist Medical Center (AHWFBMC) and Virginia Commonwealth University Massey Comprehensive Cancer Center (VCUMCCC). PALS was approved by the Institutional Review Boards at Wake Forest University (IRB00020968) and at Virginia Commonwealth University (IRB20015233).

**Fig 1:**
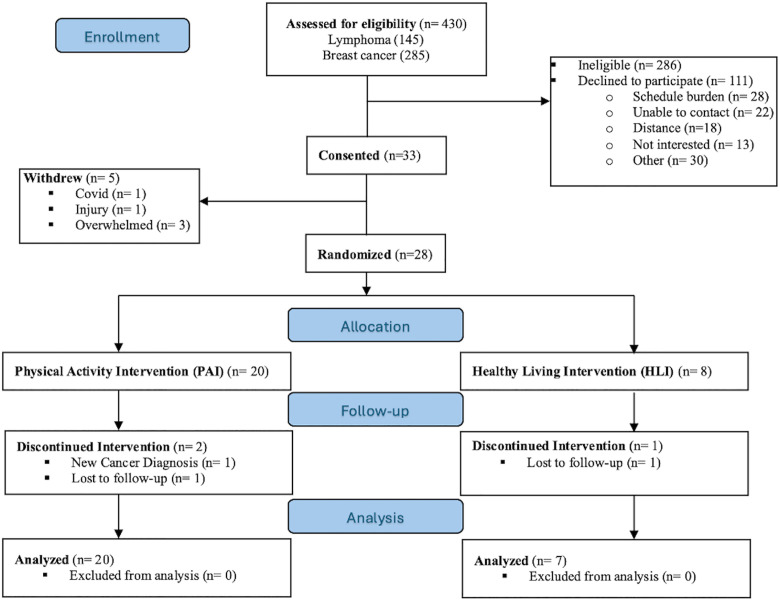
PALS CONSORT Diagram. Note: Two (2) participants who discontinued the PAI intervention were included in analyses as they had data from baseline and one other visit available. One (1) participant who discontinued the HLI intervention only had baseline data available.

In order to be considered eligible, participants had to meet the following inclusion criteria: (1) between 18 to 85 years of age, (2) diagnosed with stage I-IV Hodgkin’s or non-Hodgkin’s lymphoma, or stage I–III breast cancer, (3) expected to receive an anthracycline-based chemotherapeutic regimen or other potentially cardiotoxic cancer therapies (e.g. immuno-therapies or radiation), (4) ability to speak and understand English, (5) capacity to walk at last 2 city blocks (~0.2 miles) on a flat surface, and (6) expected to survive beyond 6 months. Individuals were excluded for having any of the following: (1) Uncontrolled hypertension (systolic blood pressure >190mmHg or diastolic blood pressure >100mmHg), (2) recent history of alcohol or drug abuse, (3) contraindications to magnetic resonance imaging (MRI), (4) claustrophobia, (5) pregnant, (6) unstable angina, (7) contraindications for exercise training or testing, (8) inability to exercise on a stationary cycle, (9) moving within 12 months of enrollment, and (10) inability to provide informed consent. Fig 1. depicts the CONSORT diagram of participant flow through the trial.

**Fig 2:**
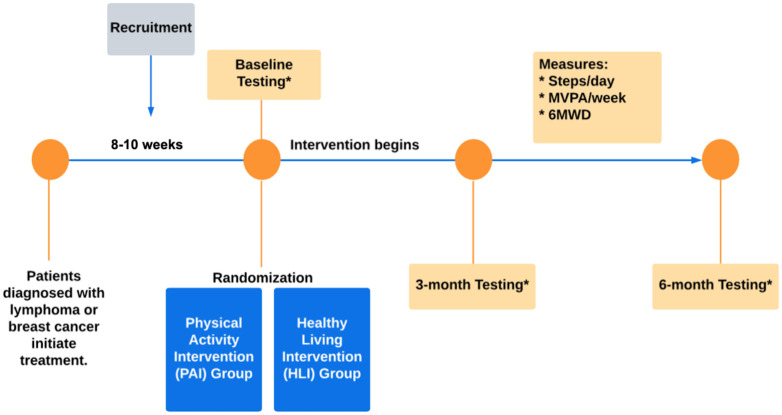
Study Schema.

Individuals were recruited at both study sites by the study’s oncologists or by a study team member via medical record review. Potential participants were interviewed to gauge interest, determine eligibility, and all participants provided written informed consent. Baseline testing was conducted as soon as possible after treatment initiation. Randomization took place following baseline testing. Fig 2. shows the study schema for the pilot trial.

Participants with breast cancer or lymphoma (n = 33) were randomized (2:1) into 6-month physical activity intervention (PAI) or healthy lifestyle intervention (HLI) groups using a variably sized permuted block randomization devised by the study biostatistician and implemented through an online clinical trials management system. Both groups wore a tri-axial accelerometer, Actigraph wGT3X-BT, on their non-dominant wrist to capture physical activity.

### Physical Activity Intervention (PAI) and Healthy Living Intervention (HLI)

The PAI was a patient-centered supervised- and/or home-based exercise training program that was tailored by treatment and functional status. In-person sessions occurred twice-weekly and were led by a clinical exercise physiologist. The PAI sessions were based on recommendations for cancer survivors from the American Cancer Society and were structured as follows: a slow (15 minute) aerobic warm up, 20 minutes of resistance training, 15 minutes of progressive intensity aerobic exercise (AE), and 10 minutes of cool-down by stretching/toning exercises. Home-based sessions, using PC or mobile devices connected to their wearable devices (i.e., Fitbit), were offered to participants unable to travel to in-person sessions and were tailored to their equipment and facilities. Due to the COVID-19 pandemic, the PAI underwent modifications that allowed participants to perform a home-based exercise training program. In-person and home-based interventions were offered once spaces reopened.

The HLI aimed to provide information on healthy lifestyle habits through didactic 60-minute bi-weekly health workshops. Interactive sessions were conducted in-person and were transitioned to Zoom because of the COVID-19 pandemic. Sessions included review of past material and introduction to new material (10 mins), an interactive Healthy Living Presentation (40 mins), and ended with instructor-led stretching exercises (10 mins). With no evidence showing that these sessions would impact the primary outcome, the HLI group served as the control group.

### Study outcome measures

The 6MWD served as a primary outcome of interest study in this study. It is a straightforward and easily attainable measure reflective of typical activities of daily living—a level of activity associated with functional independence, is highly correlated with peak exercise capacity and is an independent predictor of CV mortality [6]. The 6MWD, an objective assessments of submaximal exercise capacity (6MWD) and physical activity (steps/day, MVPA mins/week) were completed at baseline, 3-, and 6- months. Adjustments were made to allow for these measures to be collected during the COVID-19 pandemic. The Actigraph wGT3X-BT was used to gather steps/day and minutes of MVPA/week. Participants wore the Actigraph for 7 to 10 days. To be considered valid, data from at least four (≥ 4) consecutive days with ≥10 hours a day of wear time were required. Non-wear time was defined as an amplitude threshold of zero for 90 minutes, a two-minute nonzero count was permitted within the 90 minutes [7].

### Data analysis

Descriptive statistics were used to characterize the study sample. Our analytic dataset included data for 27 participants who had outcome data for at least 2 of 3 study visits. Missing data on key variables ranged between 0–28% for steps/day and MVPA mins/week, and 0–33% for 6MWD across the 3 study visits. We used a Shapiro-Wilk test to check for normality of distribution in 6MWD data by treatment group at each study timepoint and F-tests to examine homogeneity in variances prior to using an unpaired, 2-samples t-tests to examine differences in 6MWD between groups at 3- and 6-month visits. A post hoc correlational analysis examined the association between PA levels (steps/day or MVPA mins/week) and 6MWD at the 3- and 6-month timepoints in participants across both treatment arms.

## Results

**Fig 3:**
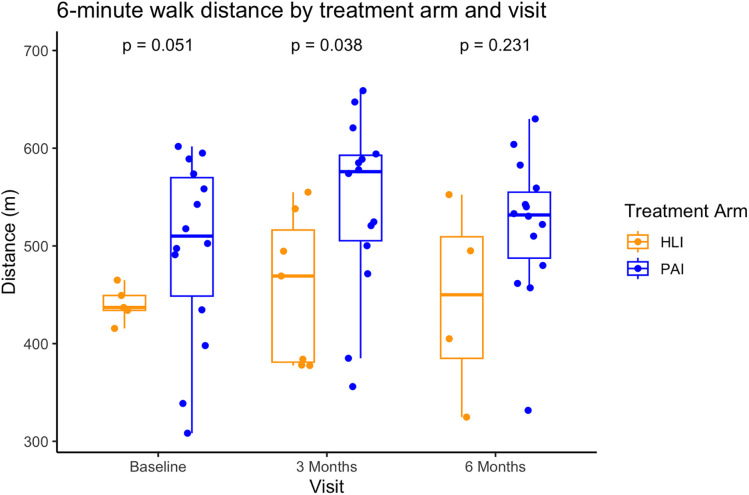
6-minute walk distance by treatment arm and visit.

Baseline demographic and clinical characteristics are shown in Table 1. At baseline, 27 participants in the PAI (n = 20) and HLI (n = 7) groups who completed baseline and at least 1 follow up visit, had a mean age of 53 (range: 23–76) vs 56 (range: 40–77) years, had been on treatment for 9.7 (range: −21.0–66.0) and 7.9 (range: −2.0–15.0) weeks, respectively. Physical activity levels in the PAI and HLI groups were 8,330 (range: 4440–14000) vs 7,800 (range: 1760–11400) steps/day, and 133 ± 44 vs 109 ± 87 minutes of MVPA/week, while their 6MWD was 496 ± 94 vs 440 ± 18 meters at baseline. Table 2 shows the descriptive data for physical activity and 6MWD by treatment arm and visit. Fig 3. shows boxplots for 6MWD by treatment arm and visit. The PAI group walked significantly further during the 6-minute walk test at 3- (87m, 95% CI 2.7–170, *p* = 0.038) but not 6-month visits (76m, 95% CI −19.3–171.2, *p* = 0.231); however, the mean 6MWD difference between groups at both timepoints is considered clinically meaningful. A correlation analysis found a significant association between steps/day (r = 0.51, p = .04) and 6MWD at 3-months but not MVPA mins/week (r = 0.48, p ≤ .051), while neither steps/day nor MVPA mins/week were associated with 6MWD at 6-months.

**Table 1:** Baseline demographic and clinical characteristics.

Mean (Range or SD)/ N (%)	PAI (N = 20)	HLI (N = 7)
**Age (years)**	52.5 (23.0-76.0)	55.9 (40.0-77.0)
**Race**		
Black	3 (15.0%)	0 (0%)
White	17 (85.0%)	5 (71.4%)
Asian	0 (0%)	2 (28.6%)
**Gender**		
Female	14 (70.0%)	3 (42.9%)
Male	6 (30.0%)	4 (57.1%)
**BMI (kg/m**^**2**^)	26.3 (10.8, 40.3)	22.4 (11.3-32.0)
**Diagnosis**		
BC	7 (35.0%)	1 (14.3%)
HL	4 (20.0%)	2 (28.6%)
NHL	9 (45.0%)	4 (57.1%)
**Time Since Diagnosis (weeks)**	35.4 (3.0-465.0)	13.6 (9.0-18.0)
**Time on Treatment (weeks)**	9.65 (−21.0-66.0)	7.86 (2.0-15.0)
**Received Anthracyclines**		
No	4 (20.0%)	3 (42.9%)
Yes	14 (70.0%)	4 (57.1%)
Missing	2 (10.0%)	0 (0%)

**Table 2:** Physical activity, and exercise capacity by treatment arm and visit.

	Baseline		3 Months		6 Months	
	PAI (N = 20)	HLI (N = 7)	PAI (N = 16)	HLI (N = 7)	PAI (N = 20)	HLI (N = 6)
**6-Minute Walk Distance (m)**						
Mean (SD)	496 (94.3)	440 (18.4)	543 (90.6)	457 (77.0)	520 (73.5)	444 (100)
Median [Min, Max]	510 [308, 602]	437 [416, 465]	576 [356, 659]	469 [378, 555]	532 [332, 630]	450 [325, 553]
Missing	6 (30.0%)	2 (28.6%)	2 (12.5%)	0 (0%)	6 (30.0%)	2 (33.3%)
**Steps/ day**						
Mean (SD)	8330 (2520)	7800 (3830)	9910 (2320)	10500 (4440)	8340 (3340)	7440 (3210)
Median [Min, Max]	8230 [4440, 14000]	8160 [1760, 11400]	9810 [5270, 13100]	12000 [5630, 15100]	8030 [1270, 15200]	7230 [4570, 12500]
Missing	3 (15.0%)	2 (28.6%)	4 (25.0%)	2 (28.6%)	0 (0%)	1 (16.7%)
**MVPA/ week**						
Mean (SD)	133 (44.2)	109 (86.9)	159 (47.3)	156 (109)	132 (56.8)	105 (76.5)
Median [Min, Max]	137 [66.8, 241]	135 [15.3, 226]	143 [78.1, 233]	186 [14.4, 267]	124 [20.8, 224]	101 [15.7, 212]
Missing	3 (15.0%)	2 (28.6%)	4 (25.0%)	2 (28.6%)	0 (0%)	1 (16.7%)

**Note:** PAI, Physical Activity Intervention; HLI, Healthy Living Intervention; MVPA, Moderate to Vigorous Physical Activity.

### Adverse events

One adverse event (AE; 2 participants) was registered as an unanticipated problem involving risks to subjects or others. This AE entailed a participant feeling nauseous and experiencing a headache after participating in an online kickboxing class provided by their local gym. The participant fully recovered within a span of 2 h. Another participant experienced a fall while walking their dog but they were not badly injured and continued with their participation in the intervention.

## Discussion

In this study, we found that the PAI group and HLI group were already close to meeting physical activity guidelines (150 minutes of MVPA) at baseline. This finding is striking given that in 2020, the CDC reported that the prevalence of meeting physical activity guidelines during leisure time in the United States was only 52% for aerobic exercise, 35% for muscle-strengthening exercise, and 28% for both [8]. The high percentage of individuals in our study meeting the physical activity guidelines suggests that those who choose to participate in physical activity intervention studies may already engage in healthier lifestyle behaviors compared to those less likely to enroll in such studies.

Surprisingly, our study also found that on average, both groups maintained their activity levels (steps/day and MVPA mins/week) up to 3 months and the PAI group slightly increased their MVPA minutes (not steps/day) by 6 months. Again, based on our baseline data, this trend may be attributed to our study sample comprising initially active individuals who might have been further motivated to adopt healthy lifestyle habits due to the interventions they received. Participants likely also started feeling better after finishing treatment contributing to the slight increase in activity levels in both groups by 6 months. It is important to note that a study conducted by van der Schoot and colleagues analyzing the optimal timing of implementing an exercise intervention in cancer patients undergoing curative chemotherapy found that VO_2_ peak did not differ between those who started a 24-week exercise intervention during chemotherapy versus after chemotherapy, whether evaluated immediately post intervention or a year after the intervention [9]. This finding supports our study as it emphasizes how, regardless of timing, an exercise prescription can benefit patient outcomes. Moreover, our finding regarding our participants’ maintenance of activity levels is remarkable given existing literature, including a systematic review finding that most patients undergoing chemotherapy are not meeting physical activity guidelines set by the World Health Organization (WHO) [10]. Additionally, data from a population-based cohort study showed that physical activity levels decline following a breast cancer diagnosis [11], likely attributed to the burden of disease and the side effects of chemotherapy including fatigue and nausea. Further, it is important to discuss that while the HLI group was not formally given an exercise prescription, healthy lifestyle habits were reviewed at workshops. With an already active group at baseline, this could suggest that educational platforms throughout treatment for already active participants could encourage and promote their established healthy habits.

Further, in both groups 6MWD was maintained. However, it is important to consider the minimal clinically important difference (MCID) when analyzing the data. The MCID represents the smallest change that makes a clinically notable and positive impact on the patient. For the 6-minute walk test, the MCID has been found to be 22–42 meters in individuals with lung cancer [12]. Another study conducted by Cantarero-Villanueva and colleagues examined individuals with only breast cancer and found the MCID for the 6MWD to be between 41m to 66m during treatment and 40m to 42m after finishing treatment [13]. Our study found that difference in 6MWD was approximately 87m and 76m further in the PAI than the HLI group at 3-and 6 months, respectively—a clinically meaningful though not statistically significant difference at 6 months. Considering the exercise prescription given to the PAI group, this may suggest that higher physical activity levels (MVPA) may associate with reduced exercise intolerance after cancer treatment. Further, our findings regarding 6MWD build on existing literature. One study randomized breast cancer patients who were undergoing treatment to either standard care or a smartphone-based multifaceted intervention emphasizing physical activity. Individuals in the smartphone group showed a significantly greater increase in 6MWD compared to those receiving standard care with an increase in 46 meters versus 8 meters (p < 0.001) [14]. Limitations of this study are 1) the missing data due to the COVID-19 pandemic and 2) that we used wrist worn accelerometers to evaluate physical activity. While this does lead to increased adherence to the wear protocol, it can also overestimate activity levels [15]. In summary, our research emphasizes the importance of physical activity during active treatments for lymphoma and breast cancer. It also underscores the importance of successfully recruiting less physically active participants, as they potentially have a greater need for such interventions and are more likely to benefit from them.

## Supporting information

S1 File PALS CONSORT 2010 Checklist. (DOCX)
